# The proteomic and metabolomic characterization of exercise-induced sweat for human performance monitoring: A pilot investigation

**DOI:** 10.1371/journal.pone.0203133

**Published:** 2018-11-01

**Authors:** Sean W. Harshman, Rhonda L. Pitsch, Zachary K. Smith, Maegan L. O’Connor, Brian A. Geier, Anthony V. Qualley, Nicole M. Schaeublin, Molly V. Fischer, Jason J. Eckerle, Adam J. Strang, Jennifer A. Martin

**Affiliations:** 1 UES Inc., Air Force Research Laboratory, Wright- Patterson Air Force Base, Ohio, United States of America; 2 The Henry M. Jackson Foundation for the Advancement of Military Medicine, Air Force Research Laboratory, Wright-Patterson Air Force Base, Ohio, United States of America; 3 Oak Ridge Institute of Science & Education, Air Force Research Laboratory, Wright-Patterson Air Force Base, Ohio, United States of America; 4 Air Force Research Laboratory, Wright-Patterson Air Force Base, Ohio, United States of America; 5 InfoSciTex Corp., Air Force Research Laboratory, Wright-Patterson Air Force Base, Ohio, United States of America; H Lee Moffitt Cancer Center and Research Institute, UNITED STATES

## Abstract

Sweat is a biofluid with several attractive attributes. However, investigation into sweat for biomarker discovery applications is still in its infancy. To add support for the use of sweat as a non-invasive media for human performance monitoring, volunteer participants were subjected to a physical exertion model using a treadmill. Following exercise, sweat was collected, aliquotted, and analyzed for metabolite and protein content via high-resolution mass spectrometry. Overall, the proteomic analysis illustrates significant enrichment steps will be required for proteomic biomarker discovery from single sweat samples as protein abundance is low in this medium. Furthermore, the results indicate a potential for protein degradation, or a large number of low molecular weight protein/peptides, in these samples. Metabolomic analysis shows a strong correlation in the overall abundance among sweat metabolites. Finally, hierarchical clustering of participant metabolite abundances show trends emerging, although no significant trends were observed (alpha = 0.8, lambda = 1 standard error via cross validation). However, these data suggest with a greater number of biological replicates, stronger, statistically significant results, can be obtained. Collectively, this study represents the first to simultaneously use both proteomic and metabolomic analysis to investigate sweat. These data highlight several pitfalls of sweat analysis for biomarker discovery applications.

## Introduction

Sweat is a biofluid that can be passively and non-invasively collected with potential links to important physiological states, such as hydration, that are known to impact human physical and cognitive performance [1]. As the push intensifies to develop wearable electronics for real-time physiological and performance-based monitoring, sweat offers an extremely attractive matrix for continuous non-invasive sample collection to fit this need. For example, integration of a real-time performance feedback mechanism, via sweat analyte monitoring, within a smart watch format would potentially provide wearers an array of information allowing for knowledge-based decision making on a personal level, such as the need for rehydration, onset of fatigue, etc. For these reasons, sweat has come to the forefront of biomarker discovery research.

Although human sweat has been studied for several decades, excreted sweat still remains an often-overlooked media source for biomarker discovery due to the relatively low abundance of analytes [2,3]. Sweat has been shown to be composed of low quantities of electrolytes, small molecules, proteins, and lipids [2–4]. The majority of sweat research has revolved around pH, chloride ions, sodium ions, potassium ions, ammonia, urea and lactate [5–27]. However, recently, discovery approaches such as mass spectrometry and NMR spectroscopy have been applied to expand our understanding of this media [28–41].

Studies on the proteomic and metabolomic content of sweat suggest both analytes are in low abundance dominated primarily by defense related proteins and amino acids [29–43]. Although relatively few proteins have been identified compared to other media sources, such as blood or tissue lysates, several groups report the potential for this media to hold proteins for biomarker discovery [29,30,34]. For example, Raiszadeh et al. show evidence for differential abundance of sweat proteins between control and schizophrenia patients [29]. Additionally, active tuberculosis has been shown to have a more diverse sweat proteome than healthy controls [34]. Similarly, sweat metabolomics has provided evidence for lung cancer diagnostics [38,39]. Additionally, the same group showed differences in metabolomic abundances between active (exercise) and passive (stimulated) sweat [36]. Collectively, these results support the hypothesis that sweat may hold proteomic and/or metabolomic biomarkers.

Establishing the link between sweat analytes and human performance will facilitate a better understanding of the mechanisms through which analytes influence and/or reflect the outcomes of performance. Furthermore, this information will allow for building predictive models of performance through which analyte abundance can be turned into ‘actionable’ information via feedback.

## Materials and methods

### Participants

The human participants (n = 11) for this study were volunteer active duty military stationed at Wright-Patterson Air Force Base (**S1 Table**). All participants were required to be between the ages of 18 to 45, have no duty restrictions, and not be on medical profile for injury or illness. All recruitment and data collection procedures were approved by the Air Force Research Laboratory Institutional Review Board (FWR2018007E) prior to initiation of the experiment. Following explanation of the experimental design, consent from each participant was obtained, including acknowledgment of the participant’s ability to leave the study at any time.

### March protocol

The exercise laboratory was monitored for temperature (n = 9, mean 22.20°C ± 0.15°C) and humidity (mean 0.2% ± 0.0%) via Kestrel 4500NV weather tracker (Minneapolis, MN, USA). Refer to **S1 Fig** for representative temperature plots.

Participants took part in two experimental sessions (A and B) separated by at least two days. The order of sessions was mixed and was completed based on participant availability. Session A: participants completed a VO_2_ max treadmill test using the Bruce protocol [44,45]. This test was used to determine participants’ aerobic capacity, ventilatory threshold, and maximum HR (bpm). Please refer to **S2 Fig** for a summary of the results. Session B: Participants were given a questionnaire to assess their regular exercise frequency and sleep duration (**S2 Fig**). Volunteers were randomly assigned to one of three test conditions: low, moderate and high intensity (**S2 Fig**). Following condition assignment, participants were equipped with sweat collection devices as outlined in the following section, and a chest-worn Polar T7 heart rate (HR) monitor (Polar Electro Inc., Lake Success, NY, USA). The monitor was placed under the standard issue Airman Battle Uniform (ABU) worn by all participants.

Participants donned approximately 22kg (48lbs) of standard issue United States Air Force (USAF) tactical gear, including a combat helmet (1.5kg, 3lb), a weighted rucksack (15.9kg, 35lb), body armor (4.5kg, 10lb), and a decommissioned M4 rifle as shown in **S2 Fig**. Once equipped, participants were instructed to march on a treadmill (Woodway, Waukesha, WI, USA) until exhaustion. Exhaustion was determined by each subject’s own perception of an exhausted state. Throughout the march, HR was monitored continuously and subjective measurements of perceived exertion were obtained using the Borg Scale every three minutes [46–48]. Please refer to **S2 Table** for march results summary. It is important to note that participants had access to water throughout the experiment.

### Sweat collection

Prior to donning the tactical gear, participants were instructed to wash their forearms with running tap water for 5–10 seconds per arm, without soap. The air-dried forearms were wiped with 70% isopropyl alcohol swabs until no visible residue was observed, and air-dried (BD, Franklin Lakes, NJ, USA). Participant’s forearms were affixed with eight adhesive free Macroduct^**®**^ Sweat Collection devices, able to retain approximately 80μL each, held in place with Velcro bands, four per arm. Compression sleeves were placed over the collection devices to keep the devices in place and induce sweat production. Refer to **S3 Fig** for representative images of collection device setup. Following collector placement, subjects donned tactical gear as outlined in the previous section.

After completion of the treadmill protocol, excreted sweat was collected from each of the eight collectors, via transfer pipette, and pooled in a single 5mL lo-bind Eppendorf tube on ice (Hamburg, Germany). The samples were immediately aliquotted, frozen on liquid nitrogen and lyophilized overnight (**S3 Fig**). Proteomics aliquots (n = 7) were supplemented with MS Safe protease inhibitor cocktail (Sigma-Aldrich, St. Louis, MO, USA). All proteomics and metabolomics (n = 10) samples were stored at -80°C until analysis.

### In-solution proteomics sample preparation

Lyophilized proteomic aliquots were resuspended in 75μL water (Optima MS Grade, Thermo Fisher Scientific, Waltham, MA, USA). Protein concentration was estimated based on the Bradford method [49]. 60μg of protein from each sample was diluted in 50mM ammonium bicarbonate (Sigma-Aldrich). Dithiothreitol (DTT, 5.6mM at 95°C for 5 minutes) reduction and iodoacetamide (10mM at ambient temperature for 30 minutes in the dark) alkylation were performed (Sigma-Aldrich). 200ng of sequencing grade modified trypsin (Promega Corporation, Madison, WI, USA) was added and samples were incubated at 37°C overnight with gentle shaking. 1μL of formic acid was added to each sample and samples were vacuum centrifuged to dryness (Pierce, Thermo Fisher Scientific). Samples were resuspended in loading buffer (2% acetonitrile: 0.03% trifluoroacetic acid (TFA, aq) and peptide concentration was estimated using a Nanodrop spectrophotometer (Nanodrop, Wilmington, DE, USA). Samples were stored at -80°C until analysis.

### In-gel proteomics sample preparation

After removal of 60μg of protein for in-solution digestion, the remainder of the proteomic samples were pooled. Two separate 14% SDS-PAGE gels were run using either 175μg of pooled sample, loading quantity based on a Nanodrop peptide estimation, or 2μg of pooled sample, loading quantity based on the Bradford analysis [49]. Proteins were fixed with 50% ethanol: 10% acetic acid for 1 hour. Gels were briefly washed with deionized water and stained overnight with BioSafe coomassie at 4°C (BioRad, Hercules, CA, USA). Stain was removed with frequent washes with MilliQ water and stored at 4°C until digestion.

Gel bands, 13 slices from the 175μg gel (Nanodrop) and 16 slices from the 2μg gel (Bradford), were excised and soaked in 50% methanol: 5% acetic acid (aq) for 1 hour twice. Please refer to **S4 Fig** for excised band locations. 200μL of acetonitrile was added for 5 minutes and gel pieces were dried in a vacuum centrifuge. Gel pieces were reduced with DTT (75μL of 32.4mM for 30 minutes at ambient temperature) and alkylated with iodoacetamide (75μL of 81.1mM for 30 minutes in the dark). Pieces were washed with 100mM ammonium bicarbonate and dehydrated with acetonitrile twice. Acetonitrile was removed in a vacuum centrifuge and gel was rehydrated for 10 minutes with 50μL of 20ng μL^-1^ of sequencing grade modified trypsin in 50mM ammonium bicarbonate. Excess trypsin solution was removed, 20μL of 50mM ammonium bicarbonate was added and samples were digested overnight at ambient temperature. Peptides were extracted by adding 30μL of 50% acetonitrile: 5% formic acid (aq) twice for 10 minutes each. Peptides were concentrated to approximately 25μL in a vacuum centrifuge. Samples were stored at -80°C until proteomic analysis.

### Proteomics liquid chromatography mass spectrometry (LC-MS/MS)

2μg of in-solution samples or 6μL of in-gel samples were injected onto a 3μ, 200Å ProntoSIL C18AQ trap column (nanoLCMS Solutions, Rancho Cordova, CA, USA) using a Dionex Ultimate 3000 RSLCnano operated in an online desalting configuration (Thermo Scientific). Peptide trapping and washing was performed isocratically using loading buffer at 5μL min^-1^ for 5 minutes. Reverse phase nano separations were performed on an Easy-Spray PepMap 50μm x 150μm, 100Å, 2μm, column at 250nL min^-1^ (Thermo Scientific). Mobile phase A consisted of 0.1% formic acid (aq) and mobile phase B consisted of 0.1% formic acid in acetonitrile (Optima MS Grade). The 180 minute analytical separation was as follows: 2% B for 5 minutes, a linear increase to 40% B at 163 minutes, 98% B wash from 165 to 168 minutes, and equilibration at 2% B from 170–180 minutes. Analytical eluent was introduced via Easy-Spray source (2.5kV) into an LTQ Orbitrap XL mass spectrometer operated in top 6 data dependent mode (Thermo Scientific). MS^1^ scans were obtained in the Orbitrap at 30,000 resolution across 350–2000 m/z. MS^n^ scans were performed in the ion trap with fragmentation occurring at 35% normalized collision energy. Dynamic exclusion settings were as follows: repeat count 3, repeat duration 30, and exclusion duration 60. In-gel samples were run under the same LC and MS conditions except analytical separations were across a 45-minute linear 2% B to 40% B gradient (64 minute total separation).

### Immunoblotting

SDS-PAGE gels were run as described in the In-gel proteomics sample preparation section. Gel proteins were transferred to nitrocellulose, Ponceau S stained, and blotted for sweat proteins. Chemiluminescent detection was performed using either the Pico or Fempto substrate (Thermo Fisher) and a GE ImageQuant RT ECL imager (Pittsburgh, PA, USA). Please refer to **S3 Table** for antibody reagent information.

### Metabolomic hydrophilic interaction liquid chromatography-mass spectrometry (HILIC-MS) analysis

Lyophilized sweat samples (n = 11) were reconstituted to aliquotted volume in 50% acetonitrile supplemented with 25nmol isotopically labeled Metabolomics Amino Acid Mix Standard (Cambridge Isotope Laboratories, Tewksbury, MA, USA). Samples were run in a randomized order (Excel, v. 14.7.7, Microsoft Corporation, Redmond, WA, USA).

Polar sweat compounds and amino acid calibration curves were separated on a Phenomenex Luna^**®**^ HILIC column (3μm, 200Å, 100 x 3mm, Torrance, CA, USA) and a Dionex UltiMate 3000 RSLC-nano utilizing the micropump at 40°C (Thermo Fisher, Waltham, MA, USA). Mobile phase A consisted of 0.01M ammonium formate (≥99.0%, Sigma-Aldrich, St. Louis, MO, USA) in 4.5% acetonitrile (aq) at pH 3.0 while mobile phase B consisted of 0.01M ammonium formate in 95% acetonitrile (aq) at pH 3.0. Injections (2μL) were subjected to the following gradient at 500μL min^-1^: 0-3min hold at 97% B, 3-9min 65% B, 9–9.5min 50% B, hold at 50% B for one minute, 10.5–11.5min 97% B and hold for 10min at 97% B. Eluent was introduced into an LTQ Orbitrap XL setup for LC-MS affixed with a heated electrospray ionization source (Thermo Fisher). For positive mode, the source and Orbitrap XL were operated with the following parameters: source voltage 4.5kV, sheath gas 8, aux gas 1, capillary temperature 275°C, and scans were made from 60-550m/z at 7500 resolution. For negative mode, the source and Orbitrap XL were operated with the following parameters: source voltage 4.5kV, sheeth gas 5, capillary temperature 280°C, and scans were made from 100-550m/z at 7500 resolution. The MS system was calibrated and tuned prior to each ionization mode run.

### Data processing

#### Proteomics

Proteomic analysis was performed using the MassMatrix search engine (v. 2.4.2) as described previously [50,51]. Briefly, tandem data was searched against the Uniprot complete H. sapiens proteome supplemented with the cRAP contaminant database using an MS^1^ mass tolerance of 10ppm, an MS^n^ tolerance of 0.8Da, and three allowed missed tryptic cleavages [52–55]. False discovery rate (FDR) was estimated using a reversed sequence database. Protein groups were required to have <5% FDR and 2 unique peptide matches to be retained in the analysis. The keratin and cRAP protein groups were removed from the analysis as contaminants but are provided in **S4 Table**. Gene ontology information, protein class, molecular function, biological process, and cellular component for identified protein groups was tabulated using the Panther Classification System (v.13.1, **S4 Table**) [56–58].

#### Metabolomics

Positive and negative metabolomics raw data files were uploaded to the XCMS Online Software Suite as a single batch for retention time alignment and feature detection [59–66]. The feature XCMS settings were as follows: centWave detection with 10ppm mass tolerance and 5-30s peak width, 1m/z orbiwarp retention time correction, and alignment bandwidth of 5s. Feature lists and abundances (161 features positive mode, 133 features negative mode) were exported for further statistical analysis.

The XCMS feature list was manually searched for [M+H]^+^ or [M-H]^-^ ions against the Metlin Database at a 5ppm mass accuracy using the simple search feature [67,68]. Searches were limited to [M+H]^+^ or [M-H]^-^ ions based on previous sweat metabolomics results [35,39]. Neat standards were ordered for the resulting tentative compound identifications. Please refer to **S3 Table** for neat standard information. Confirmatory analysis for MS/MS was performed using the HILIC methods described above. Standards and stored sample aliquots were detected in MS/MS mode on an Orbitrap Fusion Lumos mass spectrometer (Thermo Fisher). The Fusion Lumos was operated under the following conditions. For positive mode, source voltage 3.8kV, sheath gas 5.45, aux gas 2, sweep gas 3, capillary temperature 300°C, and MS^1^ scans were obtained at 60,000 resolution across a 60–300 m/z range. A mass list corresponding to neat standard m/z values was entered and fragments were generated for the mass list (±10ppm) using collision-induced dissociation (CID) at 10%, 20% and 40% normalized collision energy (10ms activation) within the ion trap (**S5 Table**). MS^2^ detection of fragment ions was performed in the Orbitrap with 7,500 resolution and a 50–300 m/z scan range over 3 microscans. For negative mode, source voltage 3.4kV, sheath gas 15, sweep gas 1, capillary temperature 300°C, and MS^1^ scans were obtained at 60,000 resolution across a 115–300 m/z range. A mass list corresponding to neat standard m/z values was entered and fragments were generated for the mass list and detected as described above (**S5 Table**).

All standard and sample MS/MS data were manually inspected and searched against the Metlin Database, as described previously, for mass accuracy and fragmentation patterns. Retention times of standards were tabulated using the XCalibur Qual browser software (v. 3.0.63, Thermo Fisher) and compared to the experimental results. Metabolomic gene ontology terms for primary process, biological role and industrial application were compiled from The Human Metabolome Database [69–72].

### Statistical analysis

Basic statistical analysis was performed using the Prism GraphPad software (v. 5.0c, GraphPad Software Inc., La Jolla, CA, USA). Additional statistical analysis was performed using the R statistical software (v.3.4.4). Metabolite abundance values were quantile normalized to account for technical variation between samples run on LC-MS at different times. Hierarchical clustering was performed on the correlation matrix of the metabolite compounds using average linkage. The resulting dendrogram was used to reorder the correlation matrix, placing most similar metabolites near one another and more dissimilar farther apart. The reordered correlation matrix was visualized with a heatmap. Similarly, the subjects were clustered based on their metabolite profiles and reordered. Generalized linear model regression analysis with LASSO regularization was performed using the glmnet package within the R software [73].

## Results and discussion

### Proteomic analysis of sweat

**Fig 1:**
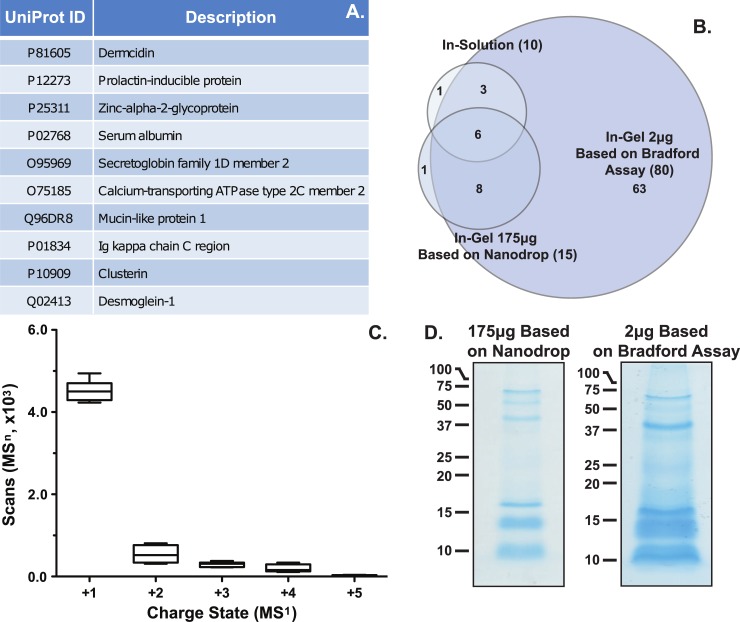
Proteomics results. A) A summary of the protein groups identified from sweat via in-solution digestion. B) A Venn diagram of the protein groups identified from in-solution and in-gel digestion. C) Box-whisker plot of the number of MS^1^ charge states selected for MS^n^ scans. D) Images of coomassie stained gels from 175μg sample loaded based on Nanodrop and 2μg sample load based on Bradford Assay. Data shows a small number of protein groups are identified from single sweat samples resulting from a high number of +1 charged peptides and a large group of low molecular weight proteins/peptides.

Historically, the proteomic analysis of sweat has yielded a wide range of protein identifications, between 95 and 861 [29–34]. The stark difference in numbers of identifications among these studies are likely due to many methodological factors, such as collection methodology, sampling locations on the body, sweat stimulation procedures, sample handling and pooling. For biomarker discovery efforts a single sample from an individual must be able to be analyzed, i.e. without pooling, with minimal preparation steps to maintain large-scale throughput. Therefore, this approach was applied to a single forearm sweat sample collected from volunteers marching on a treadmill (n = 7). Data from in-solution tryptic digestion followed by a bottom-up shotgun proteomics illustrates a low number of protein groups identified (**Fig 1A**, 5% FDR threshold and 2 unique peptides). For a complete list of protein groups, including the keratin and cRAP protein groups, identified via in-solution digestion, please refer to **S4 Table**. The low number of groups identified from individual replicates suggests additional protein and/or peptide enrichment steps are necessary to improve the overall depth obtained from a single sample [29–34]. To further support the hypothesis that the low abundance of proteins in sweat contributes to the low numbers of proteomic identifications, an in-gel tryptic digestion of pooled samples, 175μg sample load based on Nanodrop and 2μg sample load based on Bradford assay, showed an increase in the protein groups identified (80 protein groups, **Fig 1B, S4 Fig, and S4 Table**). However, the increased number of identifications were only truly observed when gels were loaded based on Bradford Assay, showing an 87.5% (10/80) increase in IDs relative to in-solution digestion, rather than loaded by Nanodrop which shows a 33.3% (10/15) increase in IDs relative to in-solution digestion (**Fig 1D**). Select protein identifications were verified by immunoblot (**S5 Fig**). Further, inspection of the combined protein groups (82), identified from both the in-solution and the in-gel analyses, suggest 96% (79/82) of the data set was previously reported in the literature with 72% (59/82) of the protein groups found by three or more research groups (**S4 Table**). While only a few novel protein groups were identified (3), these results suggest the protein groups in our study highlight that more abundant proteins in sweat may be able to be utilized for biomarker discovery. Collectively, these results suggest single replicate sweat samples are too dilute to allow for biomarker discovery without additional sample preparation strategies to enrich low abundance proteins.

**Fig 2:**
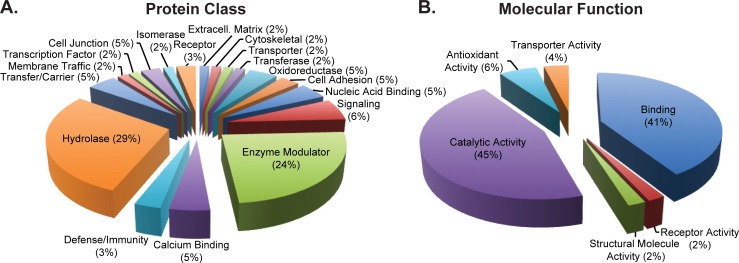
Proteomics gene ontology summary. Pie charts of the A) protein class and B) molecular function of protein groups identified from individual (in-solution) and pooled (in-gel) sweat samples. The data illustrate an enrichment of proteins associated with enzymatic activity in sweat.

Several factors beyond low protein concentration may contribute to the small number of protein groups identified via the single replicate in-solution approach. First, inspection of the raw data suggests a large number of singly (+1) charged peptides were selected for MS^n^ fragmentation (**Fig 1C**). While singly charged peptides were not excluded in the method for fragmentation, the fragments of +1 peptide ions do not generate simultaneous b and y ions from the same molecule, which can lead to difficulty in confident spectral assignment within proteomic search engines. Next, SDS-PAGE gels of pooled, 175μg sweat peptides or 2μg sweat proteins, samples show a large group of low molecular weight (<17kD) proteins or peptides are present in excreted sweat (**Fig 1D**). Generally, tryptic digests of low molecular weight proteins and peptides provide few unique peptide ions for confident protein assignment. These results would support utilization of middle-down or top-down approaches to characterize this group of proteins [32]. Next, inspection of the protein class and molecular gene ontology data illustrates the majority (53%) of the proteins fall into the hydrolase and enzyme modulator classes and 45% have catalytic activity molecular function (**Fig 2A and 2B**). Taken together, the combination of singly charged peptides, i.e. non-specific cleavage, low molecular weight proteins/peptides within the samples, and the high abundance of enzymatic protein classes suggests proteolytic degradation maybe present. However, additional research is required to define whether the degradation is due to sample handling or innate host defense, e.g. anti-microbial peptides.

### Metabolomic analysis of sweat

Similar to sweat proteomics, the metabolomic analysis of sweat has yielded a relatively small number of metabolite identifications when compared to other media sources [35–43]. Additionally, these metabolomic studies, as with the proteomic studies, utilize a diverse group of methods for collection, sweat stimulation, sample handling, and analysis [35–43]. Previous studies have illustrated a large group of polar metabolites, such as amino acids, are the predominant small molecules in this fluid [35–43]. Therefore, an untargeted metabolomics approach, using hydrophilic interaction liquid chromatography (HILIC) separation in combination with high-resolution MS detection, was applied to determine the polar metabolomic content of single sweat samples for biomarker discovery. **Table 1** shows a list of the compounds tentatively identified from single sweat samples by both positive and negative ionization modes. To verify the tentative identity of the compounds, neat standards were obtained and run for comparison of retention time and MS^n^ fragmentation patterns (**Table 1, S3 and S6 Tables**). Twenty-nine of the 48 tentative identifications (60%) were verified by retention time and/or MS/MS fragmentation (**Table 1**). Of the 48 compounds tentatively identified, 81% (39/48) had been previously reported in the literature with 54% (26/48) having four or more literature references (**S6 Table**) [35–43]. These results establish that the metabolomics approach utilized is in line with historical metabolomic analysis suggesting these are the primary metabolite targets for biomarker discovery in this media.

**Table 1:** A summary of the metabolites identified from sweat.

Compound	CAS, Metlin ID, HMDB	Median m/z	Median RT (min)	Precursor Δ Mass (ppm)	Adduct	Fragments
Urocanic Acid	104-98-3, 298, HMDB0000301	139.0499	1.11	2	[M+H]^+^	121.0398, 95.0605
		137.0363	1.13	4	[M-H]^-^	93.0457
Creatinine	60-27-5, 8, HMDB0000562	114.0659	1.14	2	[M+H]^+^	86.0961
Choline	62-49-7, 56, HMDB0000097	104.1067	1.30	3	[M+H]^+^	60.0808, 58.0647
Trolamine	102-71-6, 43365, HMDB0032538	150.1121	1.42	2	[M+H]^+^	132.1019, 114.0917
Dimethylethanolamine	108-01-0, 88280, HMDB0032231	90.0911	1.48	3	[M+H]^+^	72.0810
L-Ascorbic Acid	50-81-7, 249, HMDB0000044	175.0253	1.74	2	[M-H]^-^	-
Diolamine	111-42-2, 3239, HMDB0004437	106.0859	2.20	2	[M+H]^+^	88.0758, 70.0648
Taurine	107-35-7, 31, HMDB0000251	126.0216	2.28	2	[M+H]^+^	-
		124.0079	2.27	4	[M-H]^-^	79.9567
N-Acetyl-DL-Serine	94-14-3, 96376, HMDB0002931	146.0464	2.42	3	[M-H]^-^	74.0245
Uric Acid	69-93-2, 88, HMDB0000289	167.0215	2.60	2	[M-H]^-^	124.0149, 96.0201
L-Prolinamide	7531-52-4, 73355	115.0862	2.61	3	[M+H]^+^	70.0653
L-Phenylalanine	63-91-2, 28, HMDB0000159	166.0860	2.87	1	[M+H]^+^	120.0808
L-Leucine, L-Isoleucine	61-90-5, 24, HMDB0000687 73-32-5, 23, HMDB0000172	132.1016	2.90	2	[M+H]^+^	86.0966, 69.0692
Pyroglutamic Acid	98-79-3, 3251, HMDB0000267	128.0359	2.97	4	[M-H]^-^	82.0294
Piperidine	110-89-4, 64457, HMDB0034301	86.0962	2.99	2	[M+H]^+^	-
L-Methionine	63-68-3, 26, HMDB0000696	150.0581	3.38	1	[M+H]^+^	133.0308, 104.0518
3-Indoleacrylic acid	1204-06-4, 5702, HMDB0000734	188.0703	3.60	1	[M+H]^+^	-
L-Tryptophan	73-22-3, 33, HMDB0000929	205.0969	3.60	1	[M+H]^+^	188.0705
Pyrrolidine	123-75-1, 87832, HMDB0031641	72.0805	3.65	3	[M+H]^+^	-
L-Valine	72-18-4, 35, HMDB0000883	118.0860	3.65	2	[M+H]^+^	72.0809, 55.0539
L-Proline	147-85-3, 29, HMDB0000162	116.0703	3.70	2	[M+H]^+^	70.0653
L-Tyrosine	60-18-4, 34, HMDB0000158	182.0808	4.21	1	[M+H]^+^	165.0547, 136.0757
5-Aminopentanoic acid	660-88-8, 6902, HMDB0003355	118.0859	4.57	2	[M+H]^+^	101.0831
L-Carnitine	541-15-1, 52, HMDB0000062	162.1121	5.41	2	[M+H]^+^	-
L-Alanine	56-41-7, 11, HMDB0000161	90.0547	5.45	2	[M+H]^+^	-
Creatine	57-00-1, 7, HMDB0000064	132.0764	5.75	2	[M+H]^+^	90.0551
L-Serine	56-45-1, 30, HMDB0000187	106.0495	5.77	3	[M+H]^+^	88.0395, 60.0445
		104.0358	5.83	4	[M-H]^-^	-
L-Asparagine	70-47-3, 14, HMDB0000168	133.0605	5.77	2	[M+H]^+^	-
		131.0468	5.83	4	[M-H]^-^	113.0366
L-Glutamine	56-85-9, 18, HMDB0000641	147.0767	5.81	1	[M+H]^+^	-
Glycine	56-40-6, 20, HMDB0000123	76.0391	5.92	2	[M+H]^+^	-
5-Hydroxyectoine	165542-15-4, 63420	159.0761	6.31	2	[M+H]^+^	141.0649, 113.0712
Citrulline	372-75-8, 16, HMDB0000904	176.1027	6.31	1	[M+H]^+^	159.0765, 113.0709
		174.0890	6.34	3	[M-H]^-^	131.0825
L-Glutamate	58-86-0, 19, HMDB0000148	148.0601	6.45	2	[M+H]^+^	130.0502, 84.0437
L-Histidine	71-00-1, 21, HMDB0000177	156.0765	6.52	1	[M+H]^+^	110.0715, 83.0604
		154.0627	6.55	3	[M-H]^-^	137.0352, 93.0452
L-Aspartic Acid	56-84-8, 15, HMDB0000191	132.0307	6.62	3	[M-H]^-^	115.0035, 88.0402
L-Arginine	74-79-3, 13, HMDB0000517	175.1186	7.22	2	[M+H]^+^	70.0654, 60.0558
		173.1048	7.22	2	[M-H]^-^	131.0824
L-Lysine	56-87-1, 25, HMDB0000182	147.1125	7.36	2	[M+H]^+^	-
L-Pipecolic acid	3105-95-1, 6310, HMDB0000716	130.0859	7.36	2	[M+H]^+^	-
Ornithine	70-26-8, 27, HMDB0000214	133.0968	7.42	2	[M+H]^+^	-
		131.0831	6.34	3	[M-H]^-^	113.0366, 85.0658
L-Prolinamide	7531-52-4, 73355	115.0862	7.43	3	[M+H]^+^	-

While only 60% of the tentatively identified compounds were verified by retention time and/or MS/MS fragmentation, it is hypothesized that the tentative identifications that do not match the retention time with the neat standards may be a result of a matrix effect, such as salt content, of sweat compared to that of standards prepared in neat solutions. For example, all of the tentatively identified compounds that do not match the retention time of the neat standards do so with increased observed retention times. Increases of salt content in HILIC separations generally provide greater retention. However, it seems it may be analyte and salt dependent [74,75]. It is plausible that a matrix effect may contribute to the lack of retention time similarity among several of the tentatively identified compounds but further experimentation, such as preparing standards in representative sweat salt concentrations, will be required to confirm this hypothesis.

**Fig 3:**
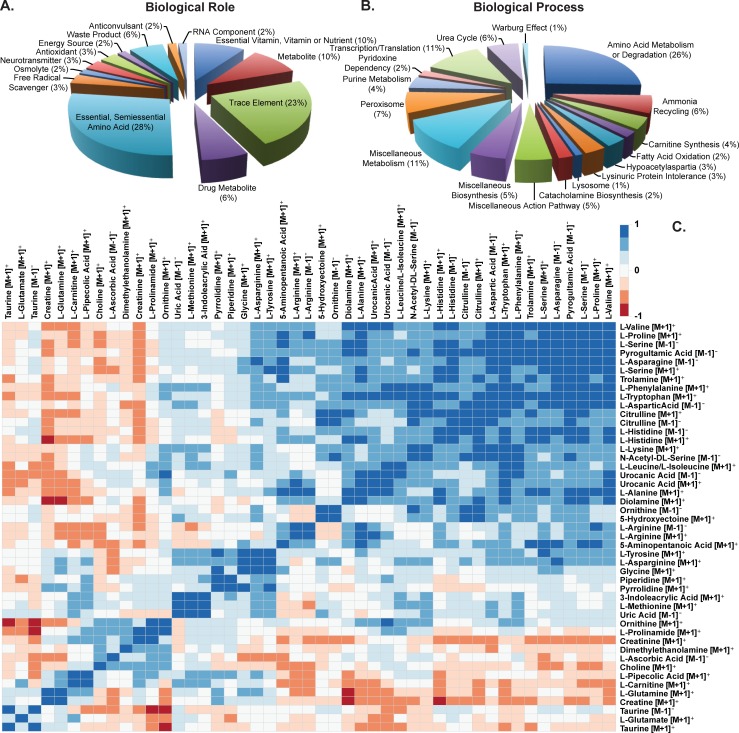
Metabolomics gene ontology and clustering of abundances. Pie charts of the A) biological role and B) biological process for metabolites identified from sweat samples. C) Clustering of metabolite abundances. Data illustrate amino acids are the primary polar metabolites present in sweat with their expression correlated among each other.

To identify the potential biological role and biological process associated with the metabolites identified, gene ontology terms were compiled from the Human Metabolome Database (**S6 Table**) [69,71,72]. Pie charts of the GO terms illustrate the largest biological role grouping is the essential and semi-essential amino acids (28%) and the predominant biological process represented is amino acid metabolism or degradation (26%, **Fig 3A and 3B**). These results support previous evidence suggesting amino acids are the most abundant metabolites in sweat [35–43]. To determine how metabolite abundances vary together, a hierarchial cluster analysis was performed. **Fig 3C** show strong correlations exist among metabolite expression profiles (**Fig 3C**). For example, a large group of metabolites tend to be increased together (**Fig 3C** lower right corner) while several others tend to decrease similarly (**Fig 3C** lower left corner). These results would illustrate a relationship among metabolite abundances in sweat.

**Fig 4:**
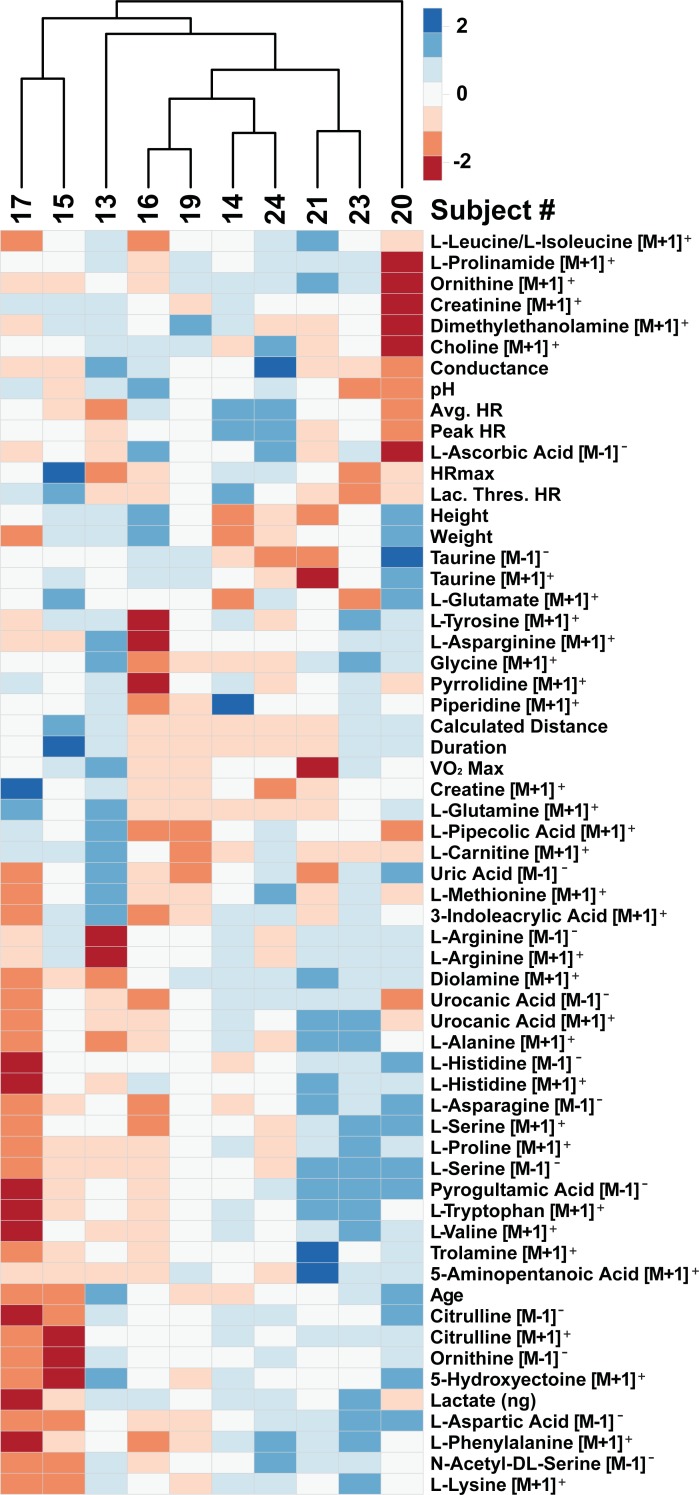
Clustering of metabolite abundances and performance measures by participant. Data illustrate participants cluster by metabolite abundance.

This investigation represents the first study simultaneously investigating both proteomics and metabolomics from the same sweat samples. Collective examination of both data sets suggests the major protein groups identified have hydrolase and catalytic activity while amino acids remain the most abundant metabolites in sweat (**Fig 2A and 2B**). Assuming hydrolase and catalytic degradation of proteins in sweat produces free amino acids lends further support for protein degradation leading to increases in free amino acids in sweat [42]. This hypothesis is further strengthened by the strong relationship in expression of the metabolites, including amino acids, in sweat (**Fig 3C**). If protein degradation were the source of amino acids in sweat, it would be expected that these metabolites would trend with exercise duration i.e. longer time for enzymes to react with proteins to make free amino acids. **Fig 4** illustrates clustering of metabolites and march parameters by participant. This limited data set shows examples of both long duration and decreased amino acids (participants 15 and 17) and long duration and increased amino acids (participants 23 and 20). Additionally, no statistically significant result was found between the metabolite abundances and physiological or march parameters. A Poisson regression was run on rate of perceived fatigue increase against the metabolites as well as linear regression of VO_2_ max against the metabolites, using LASSO variable selection (alpha = 0.8, lambda = 1 standard error via cross validation), in both cases, no metabolites were selected. While direct correlation of metabolites with physiological or performance parameters could not be accomplished, the data illustrate groupings of participant data begin to form. However, a greater number of biological replicates monitored longitudinally and more controlled experiments, accounting for potential confounding factors, will be required to truly identify metabolomic biomarkers of physiology and performance from sweat metabolomics.

As stated before, the data suggest a confounding factor or factors may exist in the metabolomics data. First, one analytical result not presented in this study is localized sweat rate. While this feature has previously been correlated with analyte concentrations, accurately estimating localized sweat rate is extremely difficult [17,23,25,76,77]. Gravimetric sweat rates, via filter paper or syringe mass changes are the most frequently published method for localized sweat rate estimation [6,7,10,15,21,22,27,78,79]. However, this method fails to take into account the latent time to initiate sweat production, which may be different depending on the individual, leading to inaccurate estimates of collection times [6,7,10,15,21,22,27,78]. Additionally, these methods ignore the excess sweat volume from saturated collection devices or incomplete recovery of sweat from within a collection pouch, yielding additional total volume. Furthermore, though several researchers have reported sweat rate, few have utilized such data for normalization of analyte concentration values [9,11,25]. Finally, others have suggested correlations between specific analytes and localized sweat rate [5,22,25]. For example, Falk et al. have observed positive correlations between lactate excretion rate in sweat and localized sweat rate in children [25]. The correlations between specific analytes and localized sweat rate must be validated, on a large scale, to be used as a normalization factor to account for sweat rate. Since the authors were uncomfortable with the accuracy of the current standard localized sweat rate determinations, sweat rate was not accounted for in this study. However, quantile normalization was applied to the metabolomics data set to attempt to remove this technical variation in abundance.

Next, the collection methodology used in this study may have contributed to some variability observed in the data set. For example, Delgado-Povedano et al demonstrated differences in sweat analytes based on collection methodology [36]. The Macroduct collection apparatus used in our study holds approximately 80μL of sweat through capture in an open-ended tube on the top of the device (**S3 Fig)**. The collector’s 80μL capacity was generally below the amount of sweat each participant yielded, suggesting an overflow of sweat lost through the open end of the collector. Therefore, the samples represent only the final portion of the exercise rather than a representation of the entire exercise. This factor may also have played a part in the confounding results for duration and metabolite abundance (**Fig 4**). As a result, investigators utilizing the Macroduct for sweat collection should be cognizant of overfilling and consider collecting the sweat prior to sample loss.

Finally, while the arms of participants were cleaned with water and isopropyl alcohol wipes, many compounds have links to industrial applications (**S6 Table**). For example, 54% (19/35) of the metabolites have been linked to personal care products (**S6 Table**). Furthermore, 60% (21/35) of the metabolites belong to the Food and Nutrition category (**S6 Table**). These results suggest further definition and experimentation surrounding the contribution of skin cosmetics/cleanliness and microbiological burden, such as comparing skin swabs prior to the experiment to the collected sweat samples, is required for further biomarker discovery from this media source.

## Conclusions

While the overall proteomic and metabolomic discovery from sweat yielded only a handful of novel identifications, this study has identified several difficulties surrounding using sweat as a medium for biomarker discovery. For example, enrichment methodologies must be optimized to concentrate low abundance protein analytes from single sweat samples. Definition of sample degradation and contamination must be outlined for proper metabolomics analysis from this medium. The data presented in this study suggest a potential for biomarker discovery from sweat. However, more controlled experiments are required to define the methodological aspects that influence the analytical results.

## Supporting information

S1 Table Participant metadata. A summary of each participant’s (n = 11) metadata.
(TIF)

S2 Table Performance results. A summary of the performance results from the march. L (Low), M (Moderate), H (High) intensity. All time is in 24-hour format.
(TIF)

S3 Table Reagents. A summary of the immunoreagents and the small molecule reagents.
(XLSX)

S4 Table Proteomics summary. A summary of the protein groups identified from the proteomic analysis of sweat including Panther gene ontology terms.
(XLSX)

S5 Table Metabolomics precursor ions. A) The m/z values used for MS/MS scan triggering in positive mode and B) in negative mode.
(TIF)

S6 Table Metabolomics summary. A summary of the metabolites identified from the untargeted metabolomic analysis of sweat including Human Metabolome Database gene ontology terms.
(XLSX)

S1 Fig Room temperature and humidity. Overlay: A plot of the mean room temperature (black circles) for each test individual (n = 10) with the overall mean (long horizontal bar, 22.20°C) and the 95% confidence interval (shorter horizontal bars, upper 22.35°C and lower 22.05°C). Underlay: A representative example of the room temperature for two individuals, one in the morning (AM) and one in the afternoon (PM), for the duration of their march. Humidity was consistent at 0.2% for all test individuals.
(TIF)

S2 Fig Questionnaire and pretesting data with experimental setup. A) A summary of the questionnaire and pretesting results. B) A summary of the test conditions and subject random assignment. C) A representative image of the march experimental setup.
(TIF)

S3 Fig Sweat collector placement and sample aliquots. A) A representative photo of the placement of the Macroduct^®^ sweat collectors B) A representative photo of the sweat collectors covered with compression sleeves. C) A summary of the volumes and aliquots from the sweat collection. Met (metabolomics), Prot (proteomics).
(TIF)

S4 Fig In-Gel results. Representations of in-gel band locations from A) 175μg sample gel based on Nanodrop (13 slices) and B) 2μg gel based on Bradford Assay (16 slices).
(TIF)

S5 Fig Verification immunoblots. Immunoblots confirming the selected proteins identified in the proteomics data set from A) individual sample replicates and B) 2μg pooled sample.
(TIF)
