# Supplementary figures and images for: The proteomic and metabolomic characterization of exercise-induced sweat for human performance monitoring: A pilot investigation
Source: PLoS One. 2018 Nov 1;13(11):e0203133. doi: 10.1371/journal.pone.0203133 (PMC6211630; doi:10.1371/journal.pone.0203133)

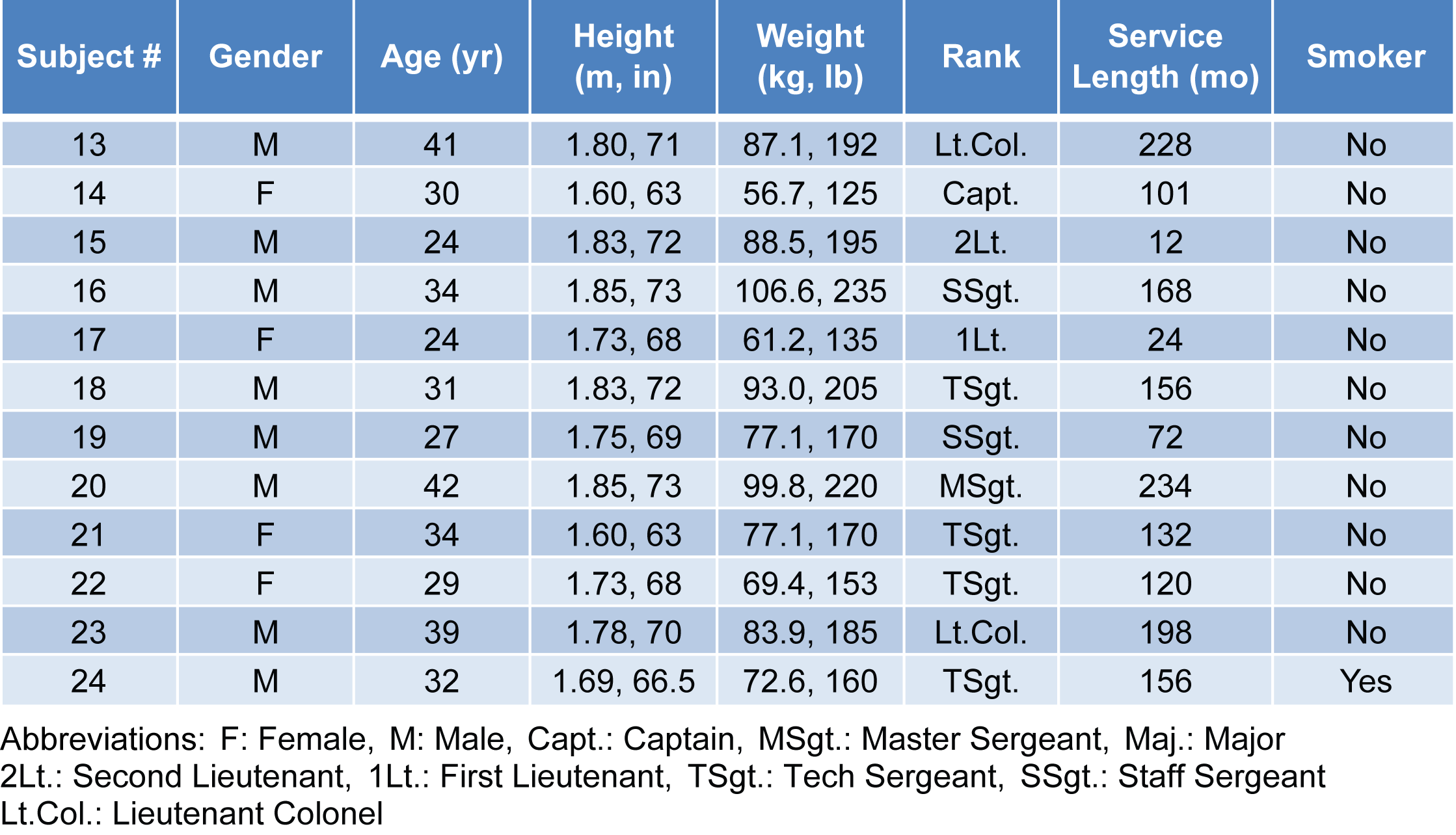

Supplement: S1 Table — A summary of each participant’s (n = 11) metadata. (TIF) [file pone.0203133.s001.tif]

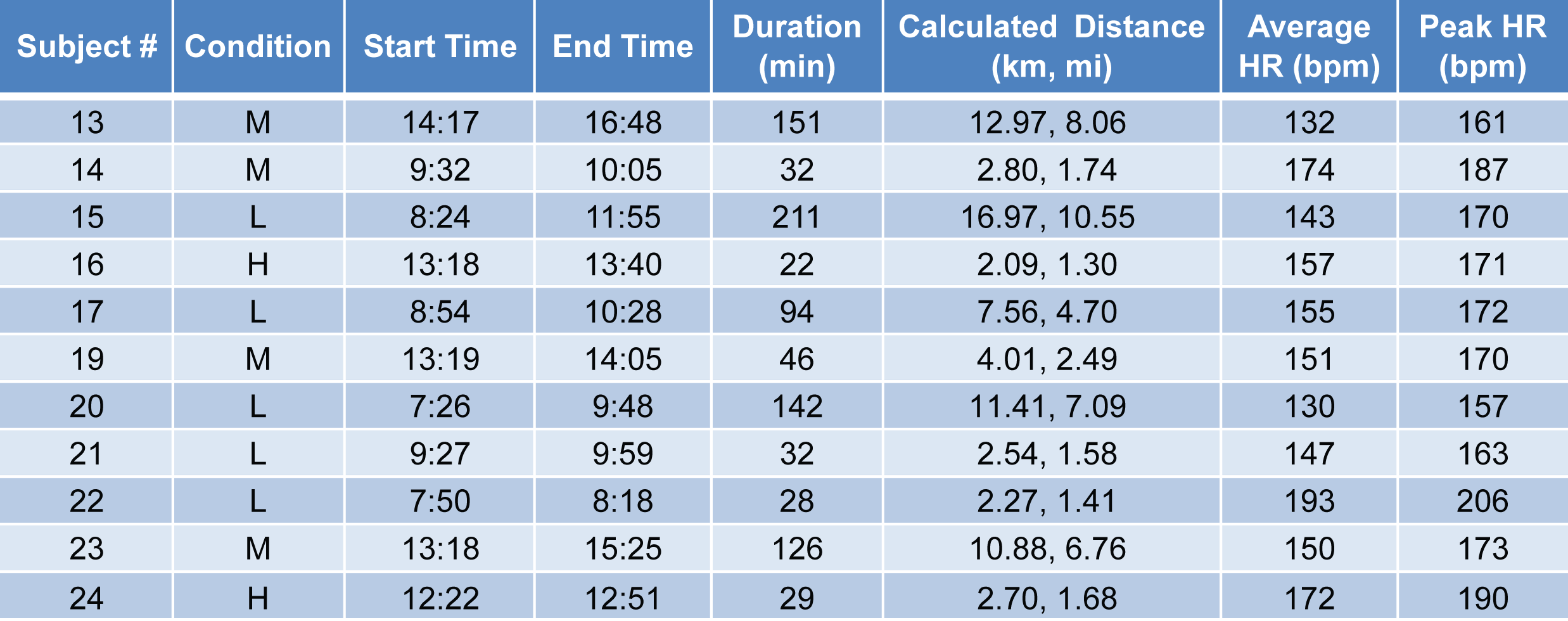

Supplement: S2 Table — A summary of the performance results from the march. L (Low), M (Moderate), H (High) intensity. All time is in 24-hour format. (TIF) [file pone.0203133.s002.tif]

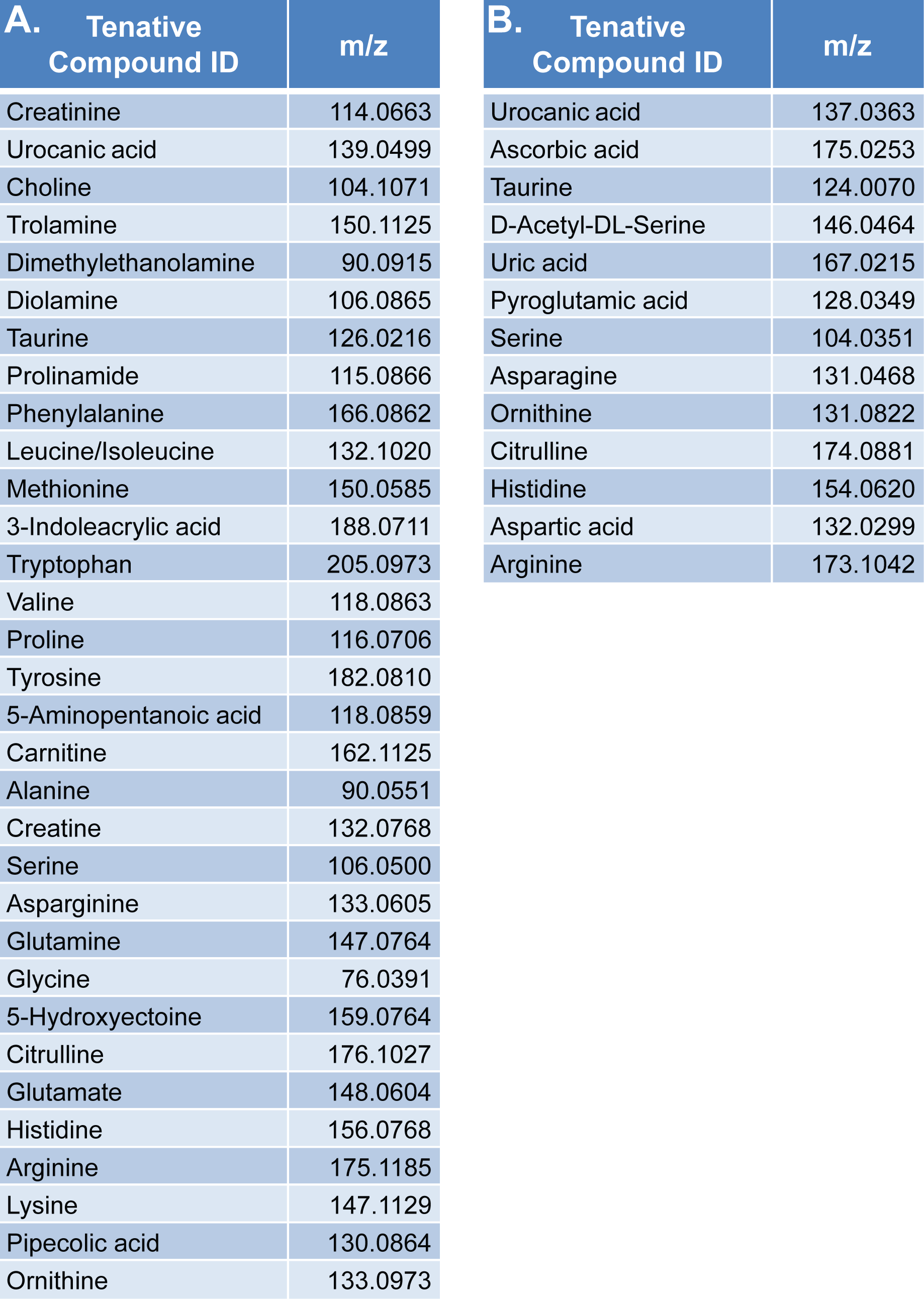

Supplement: S5 Table — A) The m/z values used for MS/MS scan triggering in positive mode and B) in negative mode. (TIF) [file pone.0203133.s005.tif]

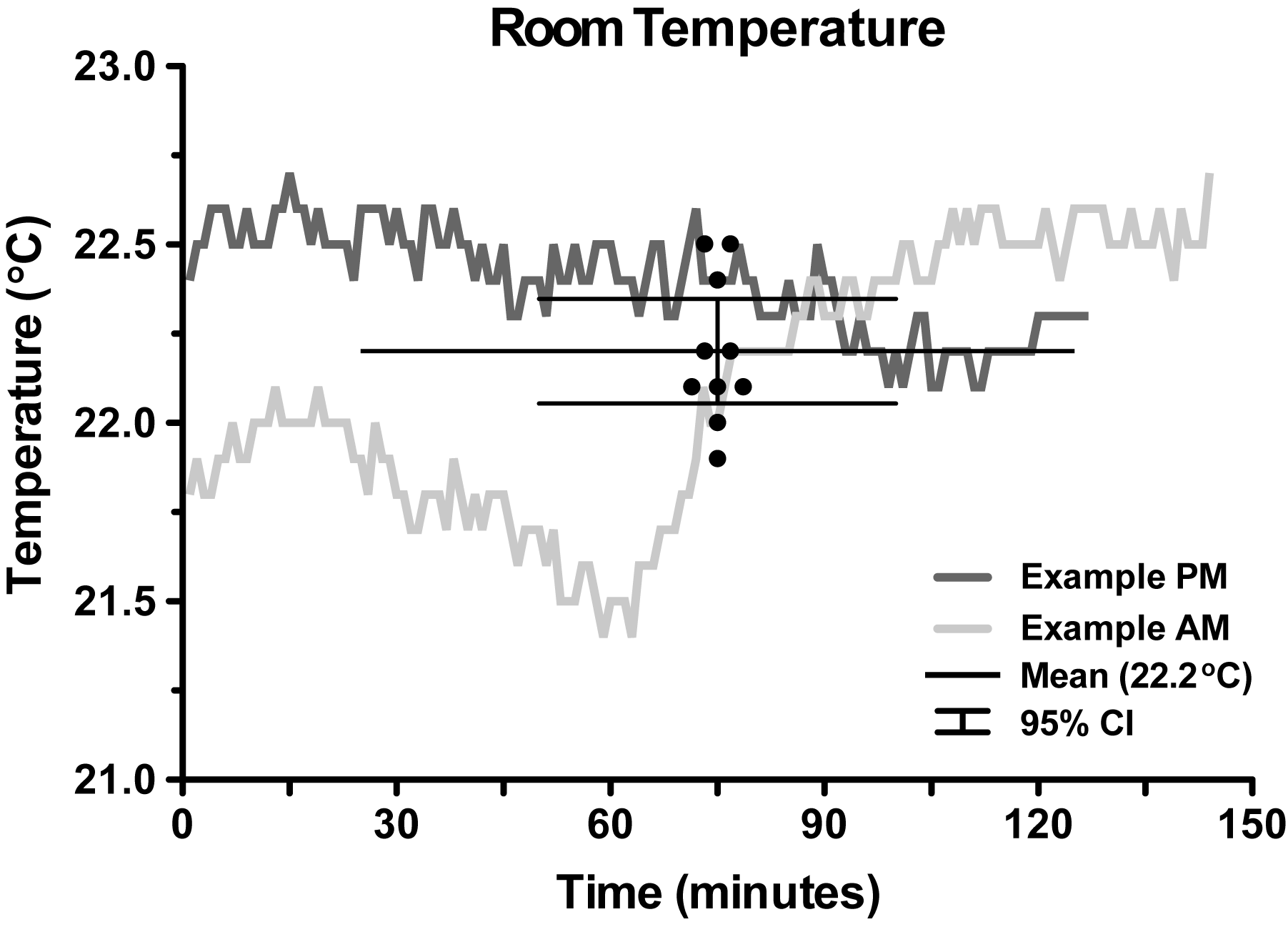

Supplement: S1 Fig — Overlay: A plot of the mean room temperature (black circles) for each test individual (n = 10) with the overall mean (long horizontal bar, 22.20°C) and the 95% confidence interval (shorter horizontal bars, upper 22.35°C and lower 22.05°C). Underlay: A representative example of the room temperature for two individuals, one in the morning (AM) and one in the afternoon (PM), for the duration of their march. Humidity was consistent at 0.2% for all test individuals. (TIF) [file pone.0203133.s007.tif]

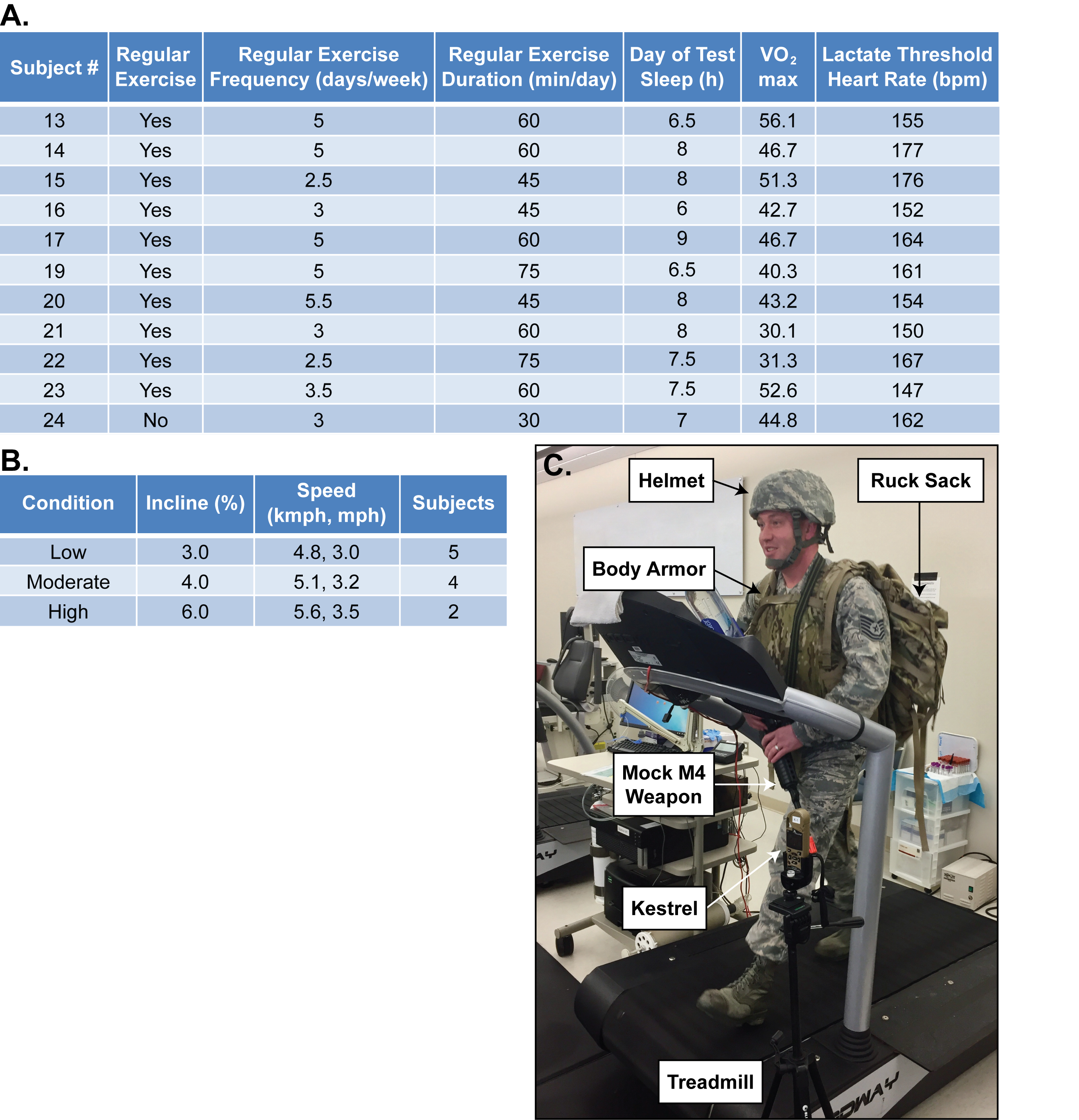

Supplement: S2 Fig — A) A summary of the questionnaire and pretesting results. B) A summary of the test conditions and subject random assignment. C) A representative image of the march experimental setup. (TIF) [file pone.0203133.s008.tif]

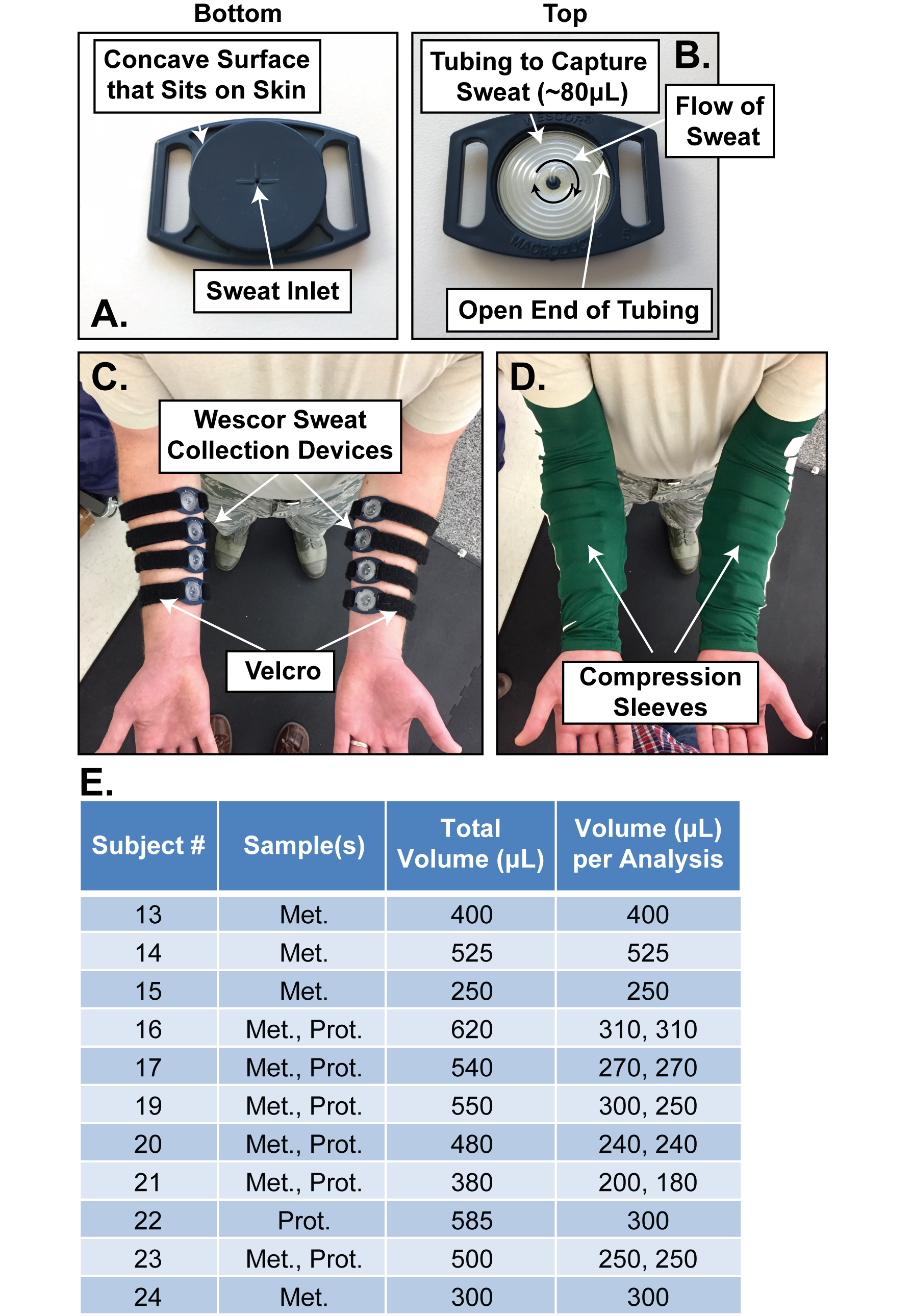

Supplement: S3 Fig — A) A representative photo of the placement of the Macroduct® sweat collectors B) A representative photo of the sweat collectors covered with compression sleeves. C) A summary of the volumes and aliquots from the sweat collection. Met (metabolomics), Prot (proteomics). (TIF) [file pone.0203133.s009.tif]

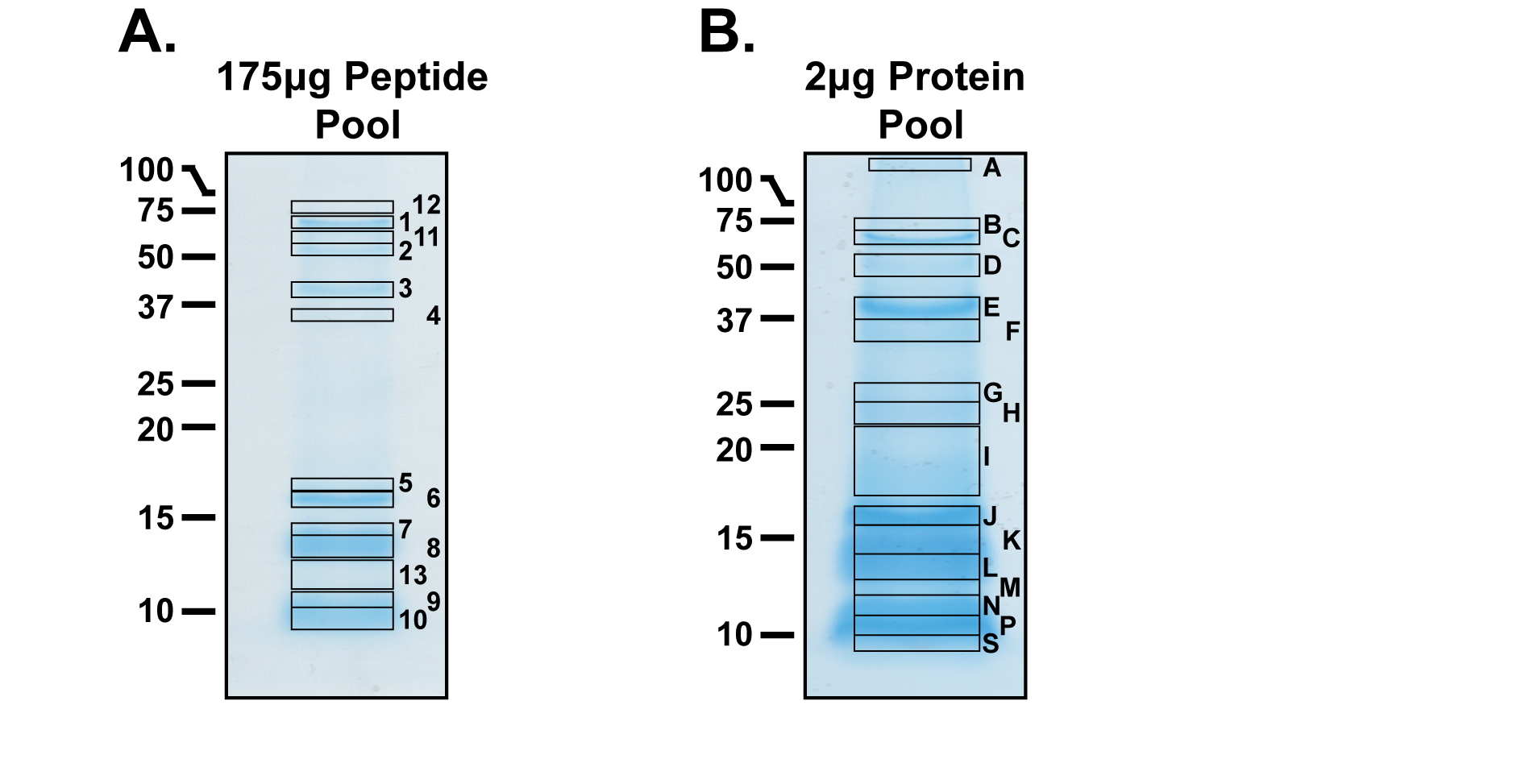

Supplement: S4 Fig — Representations of in-gel band locations from A) 175μg sample gel based on Nanodrop (13 slices) and B) 2μg gel based on Bradford Assay (16 slices). (TIF) [file pone.0203133.s010.tif]

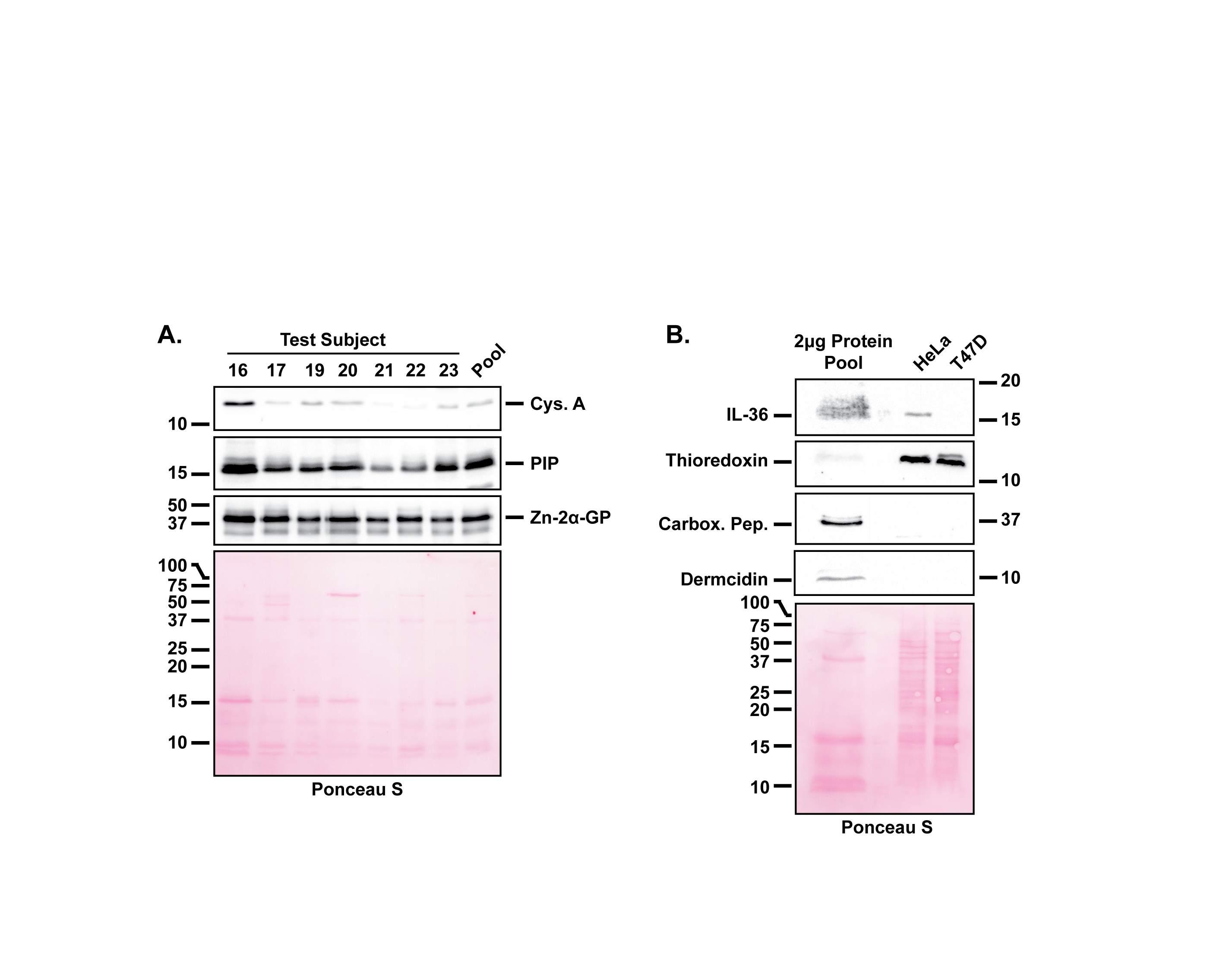

Supplement: S5 Fig — Immunoblots confirming the selected proteins identified in the proteomics data set from A) individual sample replicates and B) 2μg pooled sample. (TIF) [file pone.0203133.s011.tif]
